# Clinical and Radiological Outcomes of Intertrochanteric Femur Fractures Treated With the Trochanteric Fixation Nail: A Prospective Observational Study From a Tertiary Care Centre in South India

**DOI:** 10.7759/cureus.97909

**Published:** 2025-11-27

**Authors:** Kaarthikeyan Siva, Lionel John, Karthik Murugan

**Affiliations:** 1 Orthopaedics and Trauma, Sree Balaji Medical College and Hospital, Chennai, IND; 2 Orthopaedics, Sree Balaji Medical College and Hospital, Chennai, IND

**Keywords:** harris hip score, india, intertrochanteric fracture, radiological union, trochanteric fixation nail

## Abstract

Introduction

Intertrochanteric fractures of the proximal femur are common among elderly individuals and contribute substantially to morbidity and mortality. The trochanteric fixation nail (TFN) was designed to enhance biomechanical stability, allow early mobilisation, and minimise implant-related complications compared with extramedullary devices. However, data from Indian settings encompassing both stable and unstable intertrochanteric fractures remain limited. This study prospectively evaluated the clinical and radiological outcomes of TFN fixation across this spectrum in a tertiary care centre in South India.

Methods

A prospective observational study was conducted between January 2023 and June 2024 at a tertiary teaching hospital. Thirty adult patients with intertrochanteric fractures classified as Boyd and Griffin types I-IV underwent fixation with TFN. Data were collected using a pre-tested, semi-structured proforma encompassing operative details, radiological union, and functional outcomes assessed by the Harris Hip Score (HHS). Follow-up evaluations were performed at 1, 3, 6, and 12 months. Statistical analyses included repeated-measures analysis of variance (ANOVA) and Chi-square tests.

Results

Among 30 patients (mean age 64.2 ± 9.8 years; 17 males, 56.7%; 13 females, 43.3%), type II fractures were most common (16 patients, 53.3%). The mean operative time was 80 ± 18 minutes, and the mean intraoperative blood loss was 110 ± 25 mL. All fractures (30 patients, 100%) achieved union, with a mean radiological union time of 11.2 ± 1.8 weeks. Functional recovery improved significantly over time, with mean HHS increasing from 48.2 ± 6.4 at one month to 92.8 ± 5.2 at 12 months (F (3,87) = 64.3, p < 0.001). Complications were minimal, limited to two cases of superficial infection (two patients, 6.7%), with no implant failures or non-union.

Conclusion

TFN fixation for intertrochanteric fractures demonstrated a 100% union rate, excellent functional recovery, and a low complication profile. These findings support TFN as an effective and reliable fixation method for intertrochanteric fractures in Indian tertiary-care settings.

## Introduction

Intertrochanteric fractures of the proximal femur are among the most frequent injuries in orthopaedic trauma, particularly in elderly patients with osteoporotic bone. Their incidence is rising worldwide in parallel with ageing populations, and projections suggest that Asia, including India, will carry a disproportionate share of this increase in the coming decades [1]. These injuries are not only common but also devastating: in many cohorts, one-year mortality after hip fracture (or its surgical management) is estimated between 15% and 30%, with a frequently cited global average of ~22 [2,3], and many survivors experience a permanent decline in mobility and independence. In India, the burden is compounded by widespread osteoporosis, nutritional deficiencies, and delays in access to surgical care, leading to worse outcomes compared with high-income countries [4].

Surgery remains the cornerstone of management, aimed at providing stable fixation and permitting early mobilisation to prevent the complications of recumbency such as pneumonia, venous thromboembolism, and pressure injuries. For decades, the dynamic hip screw (DHS) was regarded as the standard implant, particularly for stable fracture patterns [5]. However, DHS has inherent biomechanical limitations in unstable fractures, including a longer lever arm and greater bending moment, which predispose to cut-out, excessive collapse, and delayed rehabilitation [6]. These shortcomings have driven the evolution of intramedullary devices, which are closer to the weight-bearing axis, require smaller incisions, and allow for earlier mobilisation [7].

Proximal femoral nails and their modifications have shown superior biomechanical stability compared with DHS, but complications such as implant migration, cut-out, and proximal femoral weakening remain concerns, especially in osteoporotic bone [8]. The trochanteric fixation nail (TFN) system was introduced to address these issues. Its helical blade provides rotational stability and improved purchase in cancellous bone while conserving native bone stock. Early reports suggest that TFN fixation offers reliable union, fewer complications, and good functional outcomes. Yet the evidence is inconsistent: many studies are retrospective, limited to Western populations, or lack adequate follow-up of functional recovery [9].

In India, the clinical context differs significantly, as patients often present late, bone quality is poorer, and rehabilitation facilities are limited. However, most published data on TFN originate from retrospective analyses or Western cohorts, where fracture epidemiology, bone quality, and rehabilitation resources differ substantially from Indian settings. To date, prospective Indian data evaluating TFN outcomes are limited, creating a gap in region-specific evidence. Addressing this gap is essential for strengthening global understanding of TFN performance across diverse populations. The present study was therefore undertaken to prospectively evaluate the clinical and radiological outcomes of intertrochanteric fractures treated with the TFN system in a tertiary care centre in South India, with a focus on fracture union, complication rates, and functional recovery assessed by the Harris Hip Score (HHS).

## Materials and methods

This study was designed as a prospective observational study that evaluated the clinical and radiological outcomes of intertrochanteric fractures treated with the TFN system. The study was conducted in the Department of Orthopaedics, Sree Balaji Medical College and Hospital, Chennai, India, which is a tertiary care centre catering to urban and semi-urban populations of South India. The study period extended over 18 months between January 2023 and June 2024, with a minimum follow-up of six months for each participant.

The sample size was calculated using the formula for repeated-measures analysis of variance (ANOVA): 

$n = \frac{2 (Z_{1-\alpha/2} + Z_{1-\beta})^{2} \sigma^{2} (1 - \rho)}{\delta^{2}}$

In this formula, n represents the required sample size per group; Z₁₋α/₂ is the standard normal deviate corresponding to a type I error of 0.05 (1.96); Z₁₋β is the standard normal deviate for 80% power (0.84); σ denotes the pooled standard deviation of the outcome measure; ρ is the correlation between repeated measures; and δ is the minimum clinically significant difference in mean outcome between time points.

Assuming σ = 15, ρ = 0.5, δ = 10, α = 0.05, and power (1-β) = 0.80, the calculated sample size was 27. Accounting for a 10% loss to follow-up, the final sample size was 30 participants.

A purposive sampling technique was adopted, and all eligible patients presenting during the study period who satisfied the inclusion criteria were enrolled consecutively until the required sample was reached. Inclusion criteria were adults aged ≥18 years, both sexes, closed intertrochanteric fractures classified as Boyd and Griffin types I-IV [10], and patients with osteoporosis. Exclusion criteria were compound or pathological fractures, previous surgery on the affected hip, and those treated with other surgical implants such as arthroplasty or alternative nailing systems.

Data were collected using a pre-tested, semi-structured questionnaire that included sections on sociodemographic details, comorbidities, mechanism of injury, fracture classification, intra-operative parameters, and post-operative outcomes (see Appendix A). The questionnaire also incorporated the HHS for functional assessment (see Appendix B) [11]. The tool was pre-tested in five patients outside the study population to ensure clarity, consistency, and feasibility, and minor modifications were incorporated before commencement. This score does not need any prior approval, and it is free to use for academic and research purposes. On admission, patients underwent detailed history taking, clinical examination, and radiographic assessment (anteroposterior and lateral views). Written informed consent was obtained. Surgery was performed under spinal or general anaesthesia with the patient supine on a fracture table. Standardised operative steps included closed or minimal open reduction, insertion of a guidewire and sequential reaming, followed by placement of the TFN (DePuy Synthes, West Chester, Pennsylvania, USA) and fixation with a proximal helical blade and distal locking screws under fluoroscopic guidance. Of the 30 procedures, 25 (83.3%) were performed using short TFN implants (180 mm), while five (16.7%) required long nails to address subtrochanteric extension or comminution extending below the lesser trochanter. Perioperative details such as blood loss, operative time, and intraoperative complications were recorded. Postoperative care comprised intravenous antibiotics for three days followed by oral antibiotics for five days, early physiotherapy, stitch removal on days 10-14, and progressive mobilisation from non-weight bearing to full weight bearing by six weeks. Patients were followed up at one, three, and six months with clinical and radiological evaluation, including range of motion, pain, deformity, union status, and HHS.

All surgeries were performed by a single orthopaedic surgeon to maintain procedural consistency. The data collectors, comprising postgraduate residents, were trained in uniform history taking, measurement of the range of motion using a goniometer, and administration of the questionnaire. Radiographs were independently reviewed by two consultants to reduce observer bias. Calibration of instruments, including goniometers and fluoroscopic machines, was verified before initiation of data collection. The data collection proforma is presented in Appendix A.

Data were entered into Microsoft Excel (Microsoft Corp., Redmond, WA, US) and subsequently analysed using IBM SPSS Statistics for Windows, Version 29 (Released 2022; IBM Corp., Armonk, New York, United States). Continuous variables were expressed as mean ± standard deviation, while categorical variables were presented as proportions. Functional outcomes were compared across time points using repeated-measures ANOVA. A p-value <0.05 was considered statistically significant. The study protocol was reviewed and approved by the Institutional Ethics Committee of Sree Balaji Medical College and Hospital (approval no. 002/SBMCH/IHEC/2023/1975). Written informed consent was obtained from all participants after explaining the nature, risks, and benefits of the study in their local language. Confidentiality was ensured by anonymising patient identifiers and reporting only aggregate data.

## Results

A total of 30 patients with intertrochanteric fractures were included in the study. Of these, 17 (56.7%) were male and 13 (43.3%) were female, with the majority of patients belonging to the 56-75 year age group (66.6%). The mean age was 64.2 years (range 45-90 years). The left hip was more commonly involved (53.3%) compared to the right (46.7%). Most fractures were sustained following low-energy mechanisms, predominantly slip and fall injuries (76.7%), while road traffic accidents accounted for the remaining 23.3%. Comorbid illnesses were present in two-thirds of patients, with diabetes mellitus (26.7%) and hypertension (23.3%) being the most frequent (Table 1).

**Table 1:** Baseline characteristics of study participants (n = 30) COPD: chronic obstructive pulmonary disease

Variable	Category	n	%
Sex	Male	17	56.7
	Female	13	43.3
Age group (years)	45-55	7	23.3
	56-65	10	33.3
	66-75	10	33.3
	76-90	3	10.0
Side of fracture	Left	16	53.3
	Right	14	46.7
Mode of injury	Fall/slip	23	76.7
	Road traffic accident	7	23.3
Comorbidities	Diabetes mellitus	8	26.7
	Hypertension	7	23.3
	Others (e.g. COPD, cardiac disease)	5	16.7
	None	10	33.3

According to Boyd and Griffin’s classification, type II fractures were the most common pattern (53.3%), followed by type IV (20%), type III (16.7%), and type I (10%) (Table 2). The mean operative duration was 80 ± 18 minutes (range 48-135 minutes), with half of the procedures completed within 60-90 minutes. Intraoperative blood loss averaged 110 ± 25 mL (range 40-170 mL). Nearly half of the patients (46.7%) lost between 100 and 150 mL of blood, while only 6.7% had minimal blood loss (<50 mL). The mean tip-apex distance (TAD) was measured on postoperative radiographs using the method described by Baumgaertner et al. [12]. All cases demonstrated a TAD <25 mm, with a mean of 22.4 ± 3.1 mm (range 16-28 mm), confirming appropriate implant positioning.

**Table 2:** Fracture classification and intraoperative parameters (n = 30)

Parameter	Value
Fracture type (Boyd and Griffin)	
Type I	3 (10.0%)
Type II	16 (53.3%)
Type III	5 (16.7%)
Type IV	6 (20.0%)
Operative time (minutes)	
30-60	3 (10.0%)
60-90	15 (50.0%)
90-120	10 (33.3%)
120-150	2 (6.7%)
Overall, mean ± SD	80 ± 18 (48-135)
Intraoperative blood loss (mL)	
<50	2 (6.7%)
50-100	9 (30.0%)
100-150	14 (46.7%)
>150	5 (16.7%)
Overall, mean ± SD	110 ± 25 (40-170)

**Figure 1:**
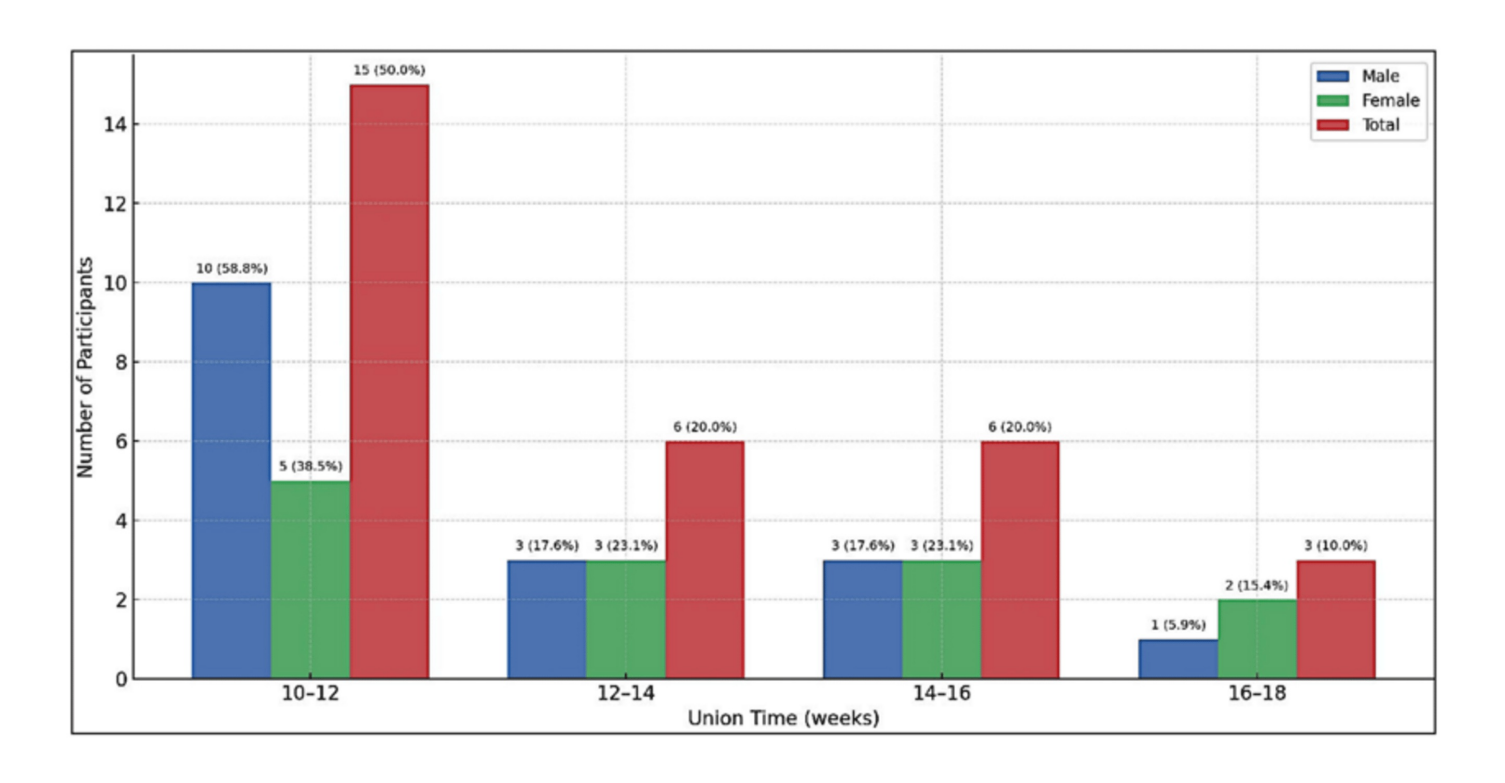
Distribution of radiological union time among study participants (n = 30)

All fractures in this series achieved radiological union. Half of the patients (50%) demonstrated evidence of union within 10-12 weeks, while an additional 40% united between 12 and 16 weeks. Only three patients (10%) required up to 16-18 weeks for complete union. The overall mean time to union was 11.2 ± 1.8 weeks (range 10-18 weeks) (Figure 1). No cases of delayed union or non-union were observed.

Functional recovery, as assessed by the HHS, demonstrated progressive improvement across all follow-up visits (Table 3). At one month, most patients (23, 76.7%) were categorised as having poor function, with a mean HHS of 48.2 ± 6.1. By three months, more than half (16, 53.3%) had improved to the fair category, with the mean HHS increasing to 62.4 ± 8.5. At six months, the majority (17, 56.7%) achieved excellent outcomes, with a mean HHS of 82.6 ± 7.4, and by 12 months, 19 patients (63.3%) were excellent and nine (30.0%) were good, with a mean HHS of 92.8 ± 5.9. Repeated-measures ANOVA revealed a statistically significant overall effect of time on mean HHS (F (3, 87) = 64.3, p < 0.001). Post-hoc pairwise comparisons with Bonferroni adjustment showed significant improvements between 1 → 3 months (p < 0.05), 3 → 6 months (p < 0.01), and 6 → 12 months (p < 0.01), confirming sustained functional recovery over the follow-up period.

**Table 3:** Functional outcomes based on Harris Hip Score (HHS) at follow-up (n = 30) Repeated-measures analysis of variance (ANOVA) showed a statistically significant within-subject effect of time on mean HHS (F (3, 87) = 64.3, p < 0.001). Post-hoc pairwise comparisons with Bonferroni correction demonstrated significant improvement in mean HHS between consecutive time points: 1 → 3 months (p < 0.05), 3 → 6 months (p < 0.01), and 6 → 12 months (p < 0.01). All p-values refer exclusively to changes in mean HHS, not to the categorical distribution of outcomes (“Poor,” “Fair,” “Good,” “Excellent”). Statistical significance was set at p < 0.05.

Time point	Poor, n (%)	Fair, n (%)	Good, n (%)	Excellent, n (%)	HHS, mean ± SD
1 month	23 (76.7)	7 (23.3)	0 (0.0)	0 (0.0)	48.2 ± 6.1
3 months	8 (26.7)	16 (53.3)	6 (20.0)	0 (0.0)	62.4 ± 8.5
6 months	0 (0.0)	6 (20.0)	7 (23.3)	17 (56.7)	82.6 ± 7.4
12 months	0 (0.0)	2 (6.7)	9 (30.0)	19 (63.3)	92.8 ± 5.9

**Figure 2:**
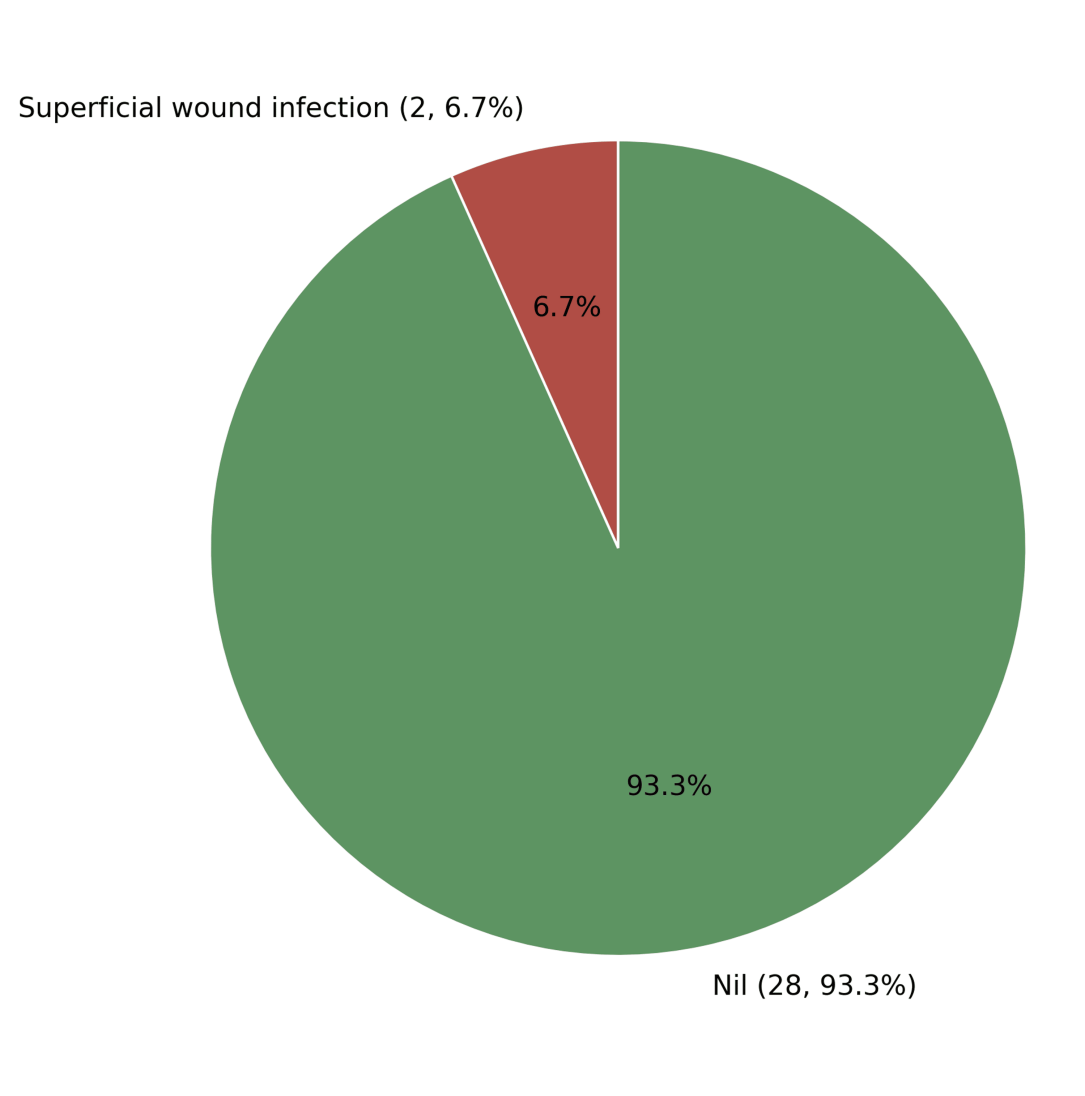
Complications observed among study participants (n = 30)

Postoperative complications were minimal. Superficial wound infection occurred in two patients (6.7%), both of whom resolved with antibiotic therapy and local care. No cases of deep infection, implant cut-out, implant failure, varus deformity, limb length discrepancy, or reoperation were encountered (Figure 2).

## Discussion

This prospective observational study evaluated the clinical and radiological outcomes of intertrochanteric fractures treated with the TFN system in a tertiary care centre in South India. Among the 30 patients studied, most sustained low-energy fractures, with type II being the most common fracture pattern. All fractures achieved radiological union, with a mean time to union of 11.2 weeks, and the majority united within 10-12 weeks. Functional outcomes, assessed by the HHS, improved steadily and demonstrated a statistically significant increase across all follow-up intervals (F (3,87) = 64.3, p < 0.001), as shown by repeated-measures ANOVA. Post-hoc pairwise analysis with Bonferroni adjustment confirmed progressive improvement between successive time points, with nearly two-thirds of patients achieving excellent scores by 12 months. Postoperative complications were minimal, limited to two cases of superficial wound infection, and no cases of implant cut-out, failure, or delayed union were reported. These findings suggest that TFN fixation provides reliable union, favourable functional recovery, and a low complication profile in the management of intertrochanteric fractures in this setting.

Comparison with existing literature

The present study demonstrates that TFN fixation of intertrochanteric fractures results in reliable union, favourable functional recovery, and minimal complications in an Indian tertiary care setting. These findings are largely in agreement with both regional and international literature, though some differences in estimates can be explained by variations in study design, fracture pattern distribution, patient demographics, and methodological rigour. Mishra et al. (2017) reported a mean union time of 11 weeks and 88% good-to-excellent HHS in 64 patients, results that align closely with the present study [13]. Similarly, Sharma and Yadav (2017) observed 83% good-to-excellent results in unstable fractures, though complication rates were slightly higher, plausibly due to the biomechanical challenges inherent in unstable patterns [14]. Reddy et al. (2021) found a mean union time of 12.7 weeks and >85% satisfactory outcomes, slightly longer than in our cohort, possibly attributable to their older patient population and stricter radiographic criteria for union [15]. Collectively, Indian studies reinforce the biological plausibility of intramedullary fixation in osteoporotic bone, where central load sharing and helical blade purchase enhance stability and reduce cut-out risk.

International meta-analyses further contextualise these results. A recent systematic review of randomised trials found that proximal femoral nails (PFN/proximal femoral nail antirotation (PFNA)) significantly reduced intraoperative blood loss and operative time compared with DHS, with no increase in nonunion or implant failure [16]. Another meta-analysis reported superior HHS, shorter fracture-healing time, and earlier weight-bearing with PFNA compared to DHS, underscoring the functional advantages of intramedullary fixation [17]. However, earlier Cochrane reviews comparing sliding hip screws with intramedullary devices found no clear superiority, noting that complication rates depended heavily on implant design, correct positioning of the sliding screw and surgical expertise [18]. The consistency of favourable outcomes in recent trials and updated meta-analyses likely reflects advances in implant design (e.g., trochanteric femoral nail-advanced (TFN-A) with helical blades) and improved surgical techniques.

Compared to Western cohorts, our findings of shorter union times and excellent functional recovery may be influenced by patient selection, relatively younger population, follow-up methodology and prevalence of type I and II fractures in this study. Western meta-analyses often include frailer, older populations with multiple comorbidities, where delayed healing and higher complication rates are expected due to reduced bone regenerative capacity and systemic fragility [19]. By contrast, our cohort included a substantial proportion of patients in their 50s and 60s, which may partly explain the faster recovery trajectories. Additionally, all surgeries in our series were performed by a single experienced surgeon, minimising technical variability, whereas multi-centre randomised controlled trials (RCTs) often report outcomes across heterogeneous surgical teams.

From a theoretical standpoint, the observed superiority of TFN is consistent with biomechanical principles: intramedullary devices reduce the lever arm and bending moment compared with extramedullary systems like DHS, translating into better resistance to varus collapse and earlier mobilisation. Current clinical practice guidelines from the American Academy of Orthopaedic Surgeons (AAOS) and the National Institute for Health and Care Excellence (NICE) recommend intramedullary fixation, particularly for unstable intertrochanteric fractures, due to these mechanical and biological advantages. This aligns with current recommendations and highlights the role of helical blade fixation as an alternative design that provides adequate purchase and rotational stability in osteoporotic cancellous bone, though not proven to be superior to conventional sliding screws when optimal positioning is achieved [20].

Clinical implications

The findings of this study have important implications. By confirming high union rates, excellent functional recovery, and a very low complication profile, our results support existing guideline recommendations (AAOS, 2021 and NICE, 2022) that intramedullary fixation should be considered the preferred treatment for intertrochanteric fractures, particularly unstable types. In low- and middle-income countries (LMICs), where healthcare resources are constrained, TFN offers additional advantages: reduced surgical exposure, lower blood loss, shorter operative duration, and earlier mobilisation. These benefits not only lower treatment costs but also reduce secondary complications such as pneumonia, pressure sores, and venous thromboembolism, which are common with prolonged immobilisation. The absence of implant cut-out in our series underscores the importance of meticulous surgical technique and optimal implant positioning, as supported by established biomechanical principles of the TFN design.

Strengths and limitations

The study has several strengths. Its prospective design with standardised operative technique by a single experienced surgeon enhances internal validity and minimises variability due to surgical learning curves. Serial radiological and functional assessments over one year provide a robust evaluation of both short- and medium-term outcomes. Furthermore, by focusing on a South Asian population with a high prevalence of osteoporosis, this study contributes region-specific evidence that complements existing Western literature and meta-analyses, thereby enhancing global generalizability. Nonetheless, certain limitations must be acknowledged. The modest sample size may restrict statistical power and limit the detection of rare complications. The single-centre design, while ensuring consistency in operative technique, may not reflect outcomes in rural or community hospitals where infrastructure and surgical expertise vary. The relatively younger age distribution of our cohort compared to Western studies may partially account for the shorter union times and better functional recovery observed, raising questions about applicability to older, frailer populations. Additionally, the absence of randomisation and exclusion of medically unfit patients introduces the potential for selection bias. Finally, the one-year follow-up, while adequate for fracture union and early functional outcomes, precludes assessment of late complications such as implant fatigue failure or long-term gait impairment. Future multi-centre, RCTs with larger and more diverse cohorts, extended follow-up, and cost-effectiveness analyses are warranted to validate and expand upon these findings.

## Conclusions

This prospective study demonstrated that the TFN achieved a 100% radiological union rate with a mean healing time of 11.2 ± 1.8 weeks. Functional outcomes improved significantly over follow-up, with nearly two-thirds of patients attaining excellent HHS by 12 months. The procedure was associated with minimal complications, limited to two superficial infections, and no cases of implant cut-out or delayed union. These findings suggest that TFN provides reliable fixation and favourable functional recovery for intertrochanteric fractures in routine tertiary-care practice. Larger multicenter studies are warranted to confirm these results and to explore implant performance across different fracture types and patient populations.

## Appendices

Appendix A

**Table 4:** Proforma for data collection

Section	Variable	Details/Options
Patient Information	Patient Name	______________________
	Age	______ years
	Sex	Male / Female
	Occupation	______________________
	IP No.	______________________
	Date of admission	___ / ___ / ______
	Date of injury	___ / ___ / ______
	Date of surgery	___ / ___ / ______
	Date of discharge	___ / ___ / ______
	Pre-operative diagnosis	______________________
	Side	Right / Left
	Classification	Boyd & Griffin (Type I–IV)
	Initial treatment details (if any)	______________________
	Associated medical illness	______________________
Present Complaints	Complaints & duration	______________________
Past History	Hypertension	Yes / No
	Diabetes Mellitus	Yes / No
	Bronchial Asthma	Yes / No
	Epilepsy	Yes / No
	Earlier injuries	Yes / No
Personal History	Smoking	Yes / No
	Alcohol	Yes / No
	Diet	Veg / Non-veg / Mixed
General Condition	Build	Normal / Thin / Obese
	Nourishment	Good / Fair / Poor
	Vitals	HR: ___, BP: ___/___, RR: ___, Temp: ___ °C
Local Examination	Side	Left ☐ / Right ☐
	Position of limb	Normal / Abnormal
	Deformity	Present / Absent
	Swelling	Present / Absent
	Tenderness	Present / Absent
	Abnormal mobility	Present / Absent
	Neurovascular deficit	Present / Absent
Preliminary Treatment	Skin traction	Yes / No
Investigations	Hb%	______ g/dL
	Blood urea	______ mg/dL
	Serum creatinine	______ mg/dL
	Blood sugar	______ mg/dL
	Blood group	______
	Total count	______
	Differential count	______
	ESR	______ mm/hr
	HBsAg	Positive / Negative
	HIV	Positive / Negative
	Chest X-ray	Normal / Abnormal
	ECG	Normal / Abnormal
Pre-op Radiological Evaluation	X-ray	Pelvis with bilateral hip AP + affected side AP/lateral
	Diagnosis	______________________
Operative Findings	Name of procedure	______________________
	Anaesthesia	Spinal / General
	Position	Supine / Other
	Reduction	Closed / Open
	Implant used	TFN / Other
	C-arm	Yes / No
	Position of implant	Satisfactory / Unsatisfactory
Post-operative Findings	Temperature	______ °C
	Dressing soakage	Yes / No
	Post-op radiological findings	______________________
Evaluation at Discharge	Suture removal	Yes / No
	Wound healing	Primary / Delayed
	Wound infection	Yes / No
	Total days of stay	______ days

Appendix B 

**Table 5:** Harris Hip Score (HHS) assessment framework Appendix B is fully adapted from the Harris WH scale, and it is cited in the reference [11]. Permission/copyright to use this scale is not required, as the HHS is publicly available and widely used in clinical research.

Domain/Sub-domain	Option/Criterion	Points
Pain (Maximum 44)	None or ignores it	44
	Slight, occasional, no compromise in activities	40
	Mild pain; no effect on average activities; may take aspirin	30
	Moderate pain; tolerable, some limitation; occasional stronger analgesics	20
	Marked pain; serious limitation	10
	Totally disabled; bedridden	0
Limp (Maximum 11)	None	11
	Slight	8
	Moderate	5
	Severe	0
Support	None	11
	Cane for long walks	5
	Cane full time	2
	Crutch	3
	Two canes	2
	Two crutches	1
	Unable to walk	0
Distance Walked	Unlimited	12
	Two or three blocks	6
	Six blocks	4
	Indoors only	2
	Bed and chair only	0
Activities	Sitting – Any chair 1 hour	6
	High chair	4
	Unable to sit 30 min in any chair	0
	Public transport – Able to enter	5
	Unable to use	0
	Stairs – Normally without railing	3
	Normally with railing	2
	In any manner	1
	Unable to do stairs	0
	Shoes & Socks – With ease	5
	With difficulty	2
	Unable	0
Range of Motion (ROM)	Flexion (normal 140°): _____°	
	Abduction (40°): _____°	
	Adduction (40°): _____°	
	External rotation (40°): _____°	
	Internal rotation (40°): _____°	
	Total ROM (°)	Points
	211–300	5
	161–210	4
	101–160	3
	61–100	2
	31–60	1
	0–30	0
Absence of Deformity (Maximum 4)	Flexion contracture <30°	☐ Yes ☐No
	Fixed abduction <10°	☐ Yes ☐No
	Fixed internal rotation <10°	☐ Yes ☐No
	Limb length discrepancy <3.2 cm	☐ Yes ☐No
Total Harris Hip Score (HHS) Calculation	Pain	____ / 44
	Function—Gait (Limp + Support + Distance)	____ / __
	Function—Activities	____ / __
	Range of Motion	____ / 5
	Absence of Deformity	____ / 4
Total HHS		____ / 100
Interpretation	Poor < 70 Fair 70–79 Good 80–89 Excellent 90–100	
